# A Novel Piecewise Frequency Control Strategy Based on Fractional-Order Filter for Coordinating Vibration Isolation and Positioning of Supporting System

**DOI:** 10.3390/s20185307

**Published:** 2020-09-16

**Authors:** Yeying Tao, Wei Jiang, Bin Han, Xiaoqing Li, Ying Luo, Lizhan Zeng

**Affiliations:** State Key Laboratory of Digital Manufacturing Equipment and Technology, Huazhong University of Science and Technology, Wuhan 430074, China; taoyy@hust.edu.cn (Y.T.); Jiangw@hust.edu.cn (W.J.); binhan@hust.edu.cn (B.H.); xqli@hust.edu.cn (X.L.); ying.luo@hust.edu.cn (Y.L.)

**Keywords:** piecewise frequency control, coordinating vibration isolation and positioning, direct and external disturbances, Fractional-Order Filter, constrained nonlinear multivariable function

## Abstract

A piecewise frequency control (PFC) strategy is proposed in this paper for coordinating vibration isolation and positioning of supporting systems under complex disturbance conditions, such as direct and external disturbances. This control strategy is applied in an active-passive parallel supporting system, where relative positioning feedback for positioning and absolute velocity feedback for active vibration isolation. The analysis of vibration and deformation transmissibility shows that vibration control increases low-frequency position error while positioning control amplifies high-frequency vibration amplitude. To overcome this contradiction across the whole control bandwidth, a pair of Fractional-Order Filters (FOFs) is adopted in the PFC system, which increases the flexibility in the PFC design by introducing fraction orders. The system stability analysis indicates that the FOFs can provide a better stability margin than the Integral-Order Filters (IOFs), so the control gains are increased to get a better performance on the AVI and positioning. The PFC based on FOFs can suppress the peak amplitude at the natural frequency which cannot be avoided when using the IOFs. The constrained nonlinear multivariable function is formed by the required performance and the stability of the system, then the controller parameters are optimized effectively. Lastly, the effectiveness of the proposed method is verified by experiments.

## 1. Introduction

Sensitive units of precision instruments such as atomic force microscopes [1], photolithography [2], electro-optical systems [3] need to maintain their stable positions under complex disturbances. The spring-damper supporting system is the most commonly used technique for suppressing disturbances. This technique can isolate high-frequency disturbances from the base, but the vibration amplitude is amplified around the inherent frequency. Moreover, due to the low supporting stiffness [4,5], the passive supporting system is easily deformed by disturbances, which leads to the leveling problem. As the counterpart, the active vibration isolation (AVI) system can make up for these deficiencies [6,7].

The dynamic characteristics of the AVI system can be adjusted through various feedback controls. The virtual supporting stiffness can be reduced by the positive relative position feedback (RPF) control [5] and the virtual mass can be increased by the acceleration or force feedback control [8]. Thus, the inherent frequency of the supporting system can be reduced to improve vibration isolation bandwidth [9]. The relative damping can be obtained through the relative velocity control, which attenuates the vibration around the inherent frequency with the cost of weakening the ability of the high-frequency vibration isolation [10]. Using the absolute velocity feedback (AVF) control or the integral force feedback control [11], the absolute damping can be obtained to address this problem. In addition, by increasing the virtual stiffness, the position accuracy, and the resistance to direct disturbances are improved [12]. However, if the support system experiences complex disturbances, it is necessary to effectively synthesize multiple types of feedback controls.

**Figure 1:**
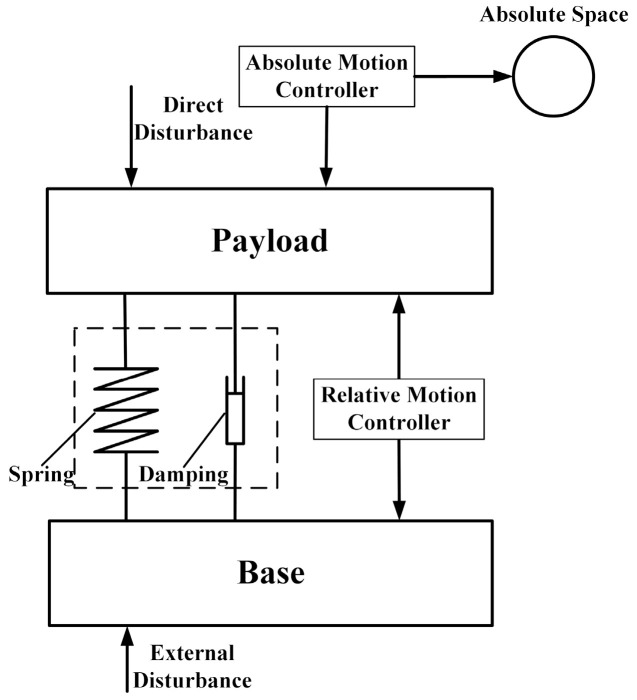
The theory of active vibration isolation (AVI) and positioning.

To enhance the positional stability of the supporting system, multiple types of feedback control composed of the absolute motion feedback and the RPF are widely used. To ensure the AVI performance and the position stability, Liao et al. [13] used the AVF and the RPF to obtain the sky-hook damping and improve the relative position stability, respectively. Beijen et al. [14] added the virtual mass and skyhook damping using the proportional and integral (PI) acceleration feedback control and contributed to the virtual stiffness through the RPF for a soft-mounted vibration isolator. In microgravity environments, the acceleration feedback and the RPF are also employed simultaneously. Zhang et al. [15] employed the position control loop to keep the contactless actuator from crashing and employed the acceleration control loop to eliminate the vibration of the payload for the MAIS (Microgravity active vibration isolation system). The g-limit (Glovebox Integrated Microgravity Isolation Technology) [16] was also controlled by these two control loops to cancel the inertial motion and keep the platform centered. Similarly, the force feedback combined with the RPF also has the same control effect. In a flexible stewart platform, Yang et al. [17] compensated for the stiffness of the flexure hinge using the position feedback control and employed the PI force feedback to broaden the vibration isolation bandwidth and add to the absolute damping. To resist the disturbances during the positioning process, Yun et al. [18] combined the position control loop and acceleration control loop in the compliant parallel manipulator to achieve vibration attenuation and micro-positioning. As shown in Figure 1, it is worth considering the fact that the RPF tightens the connection between the payload and the base, which weakens vibration isolation. Setting the relative position regulation as the secondary control objective, a low-frequency RPF was adopted in the micro-gravity vibration isolation system [19] and the advanced LIGO (laser interferometer gravitational-wave observatory) [20], while the low-frequency RPF with the cut-off frequency of 1 Hz was used in the HT (Hood Technology Corporation)/UW (University of Washington) hexapod [21]. However, the performance of the AVI and positioning was discussed separately or only one of these factors was discussed by the above scholars. The interaction between the AVI and positioning is still lacking the in-depth research needed to achieve effective coordination.

To avoid the undesired effects of the feedback controllers on the frequency range, the Integral-Order Filter (IOF) is adopted to limit the bandwidth of the feedback signals. However, the magnitude and phase margin may be reduced by inserting the IOF into the control system, which means that the system tends to be unstable. In recent years, the Fractional-Order Filter (FOF) has been increasingly used in dynamic systems [22,23,24], which provides greater flexibility to optimize both the magnitude and phase responses in comparison with the IOF [25]. Though the analysis of the mutual restriction between vibration and deformation transmissibility, a novel piecewise frequency control (PFC) system based on FOFs is presented in this paper to effectively weaken the contradiction between the AVI and positioning of the supporting system. The FOFs can provide a larger stability margin than the IOFs, so the control gains can be increased to get a better performance on the AVI and positioning. In Section 2, the PFC strategy for the vibration isolation and positioning (VIP) system is described and the interaction between the AVI and positioning is explained. In Section 3, the influence of the AVF and RPF on the supporting system is analyzed. In Section 4, the PFC based on FOFs is analyzed and optimized. In Section 5, the experimental setup is described, and the proposed control approach is verified by the experimental results. In Section 6, the conclusions are presented.

## 2. Description of PFC Strategy for VIP

As shown in Section 1, the complex disturbances affect the sensitive units of precision instruments, resulting in loss of accuracy. The VIP system is applied to restrain the disturbances to an acceptable level and enhance the positional stability, which can commonly be described as an active-passive parallel supporting system. The active component of the VIP system consists of actuators, sensors, and a control system. The signals collected from relative position sensors and absolute motion sensors are transmitted to the actuator for the AVI and positioning in the VIP system. To focus on the essence of the coordinated control problem, the analysis presented in this paper is limited to a single degree of freedom (DOF) VIP system.

**Figure 2:**
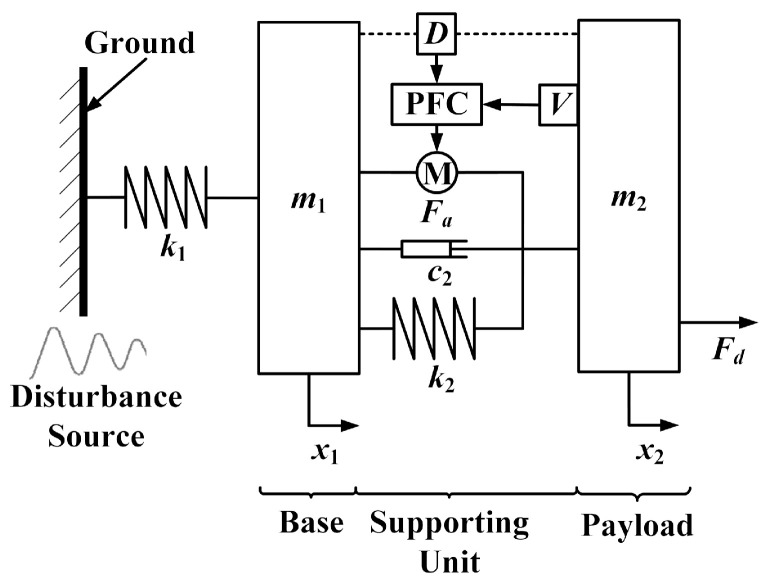
Single-degree of freedom (DOF) supporting systems with a piecewise frequency control (PFC) strategy for vibration isolation and positioning (VIP).

**Figure 3:**
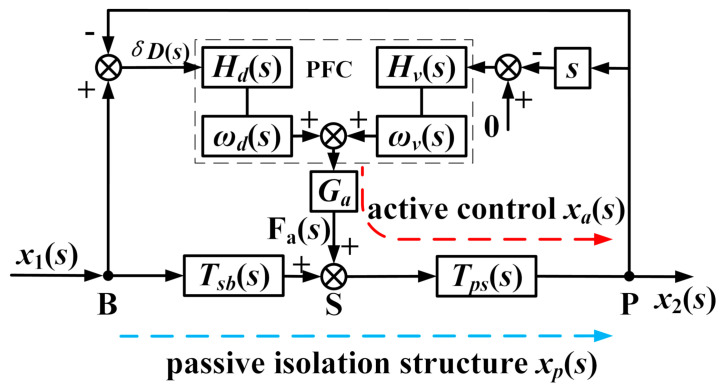
Block diagram of the PFC strategy for VIP. The PFC is shown in the dotted box. The external disturbance *x*_1_ is transmitted to point P by the active control loop, shown as the red dotted line, for which the response motion is *x_a_*; by the passive isolation structure, shown as the blue dotted line, for which the response motion is *x_p_*.

As shown in Figure 2, the VIP system is composed of a passive supporting unit and an active control system. The payload and the base are modeled as two rigid bodies, i.e., the masses *m*_2_ and *m*_1_, respectively. They are interconnected by the passive supporting unit consisting of the stiffness *k*_2_ and the damping *c*_2_. The disturbance is transmitted from the ground to the mass *m*_1_ through the stiffness *k*_1_ to produce the external disturbance *x*_1_. The payload position *x*_2_ is disturbed by an external disturbance *x*_1_ and a direct disturbance *F_d_*. The passive supporting unit can sufficiently attenuate high-frequency external disturbances *x*_1_, but this amplifies the vibrations around its inherent frequency and limits its ability to suppress the direct disturbance *F_d_*. In the active control system, a velocity sensor located on the payload is used to measure the absolute velocity of the payload, and an eddy-current sensor positioned in parallel with the supporting unit is used to obtain the relative position *δD* between the payload and the base. The actuator M is a voice coil motor (VCM) that is used to produce a force *F_a_* according to the AVI and positioning control. As shown in Figure 3, to attenuate the absolute velocity *x_2_* and maintain the relative motion *δD*, the PFC is composed of the AVI controller *H_v_*(*s*), the positioning controller *H_d_*(*s*), and the filters *ω_v_*(*s*) and *ω_d_*(*s*).

To describe the PFC strategy, the transfer function of the passive isolation structure is written as

(1) $$T_{pb}\left(s\right)=\frac{x_{p}\left(s\right)}{x_{1}\left(s\right)}=\frac{c_{2}{s+k}_{2}}{m_{2}s^{2}{+c}_{2}{s+k}_{2}},$$

where *x_p_*(*s*) is the payload motion caused by the passive isolation structure transmitting the base motion *x*_1_(*s*). As the connection stiffness *k*_1_ can be considered infinite, the transfer function *T_ps_*(*s*) can be obtained by

(2) $$T_{ps}\left(s\right)=\frac{x_{a}\left(s\right)}{F_{a}\left(s\right)}\approx \frac{1}{m_{2}s^{2}{+c}_{2}{s+k}_{2}},$$

where *x_a_* is the payload motion caused by the actuator force *F_a_*. Thus, the transfer function *T_sb_*(*s*) can be expressed as

(3) $$T_{sb}\left(s\right)=\frac{T_{pb}\left(s\right)}{T_{ps}\left(s\right)}\approx c_{2}{s+k}_{2}.$$

From Figure 3, the motion equation of the VIP system resisting the external disturbance *x*_1_ can be represented by

(4) $$\begin{array}{ll} x_{2}\left(s\right) & =\left(F_{a}\left(s\right)+x_{1}\left(s\right)T_{sb}\left(s\right)\right)T_{ps}\left(s\right)\\ & =F_{a}\left(s\right)T_{ps}\left(s\right)+x_{1}\left(s\right)T_{pb}\left(s\right). \end{array}$$

Because the AVI is the negative feedback of the velocity *x_2_s*, which is used to suppress the vibration velocity. And the positioning control is used to maintain the relative position *δD* between *m*_1_ and *m*_2_. Considering the VCM as a gain *G_a_*, the actuator force *F_a_* is given as

(5) $$\begin{array}{ll} F_{a}\left(s\right) & =G_{a}\left(H_{d}\left(s\right)\omega_{d}\left(s\right)\delta D\left(s\right)-H_{v}\left(s\right)\omega_{v}\left(s\right)sx_{2}\left(s\right)\right)\\ & =G_{a}\left(H_{d}\left(s\right)\omega_{d}\left(s\right)x_{1}\left(s\right)-\left(H_{v}\left(s\right)\omega_{v}\left(s\right)s+H_{d}\left(s\right)\omega_{d}\left(s\right)\right)x_{2}\left(s\right)\right). \end{array}$$

Then, by substituting the value of *Fa* from Equation (5) into Equation (4), the performance of the AVI and positioning can be evaluated by the vibration transmissibility *T_iso_* (*s*):

(6) $$T_{iso}\left(s\right)=\frac{x_{2}\left(s\right)}{x_{1}\left(s\right)}=\frac{T_{pb}\left(s\right)+G_{a}H_{d}\left(s\right)\omega_{d}\left(s\right)T_{ps}\left(s\right)}{{1+G}_{a}\left(H_{d}\left(s\right)\omega_{d}\left(s\right)+H_{v}\left(s\right)\omega_{v}\left(s\right)s\right)T_{ps}\left(s\right)},$$

and by the deformation transmissibility *T_pos_*(*s*):

(7) $$T_{pos}\left(s\right)=\frac{\delta D\left(s\right)}{x_{1}\left(s\right)}=\frac{1+G_{a}H_{v}\left(s\right)\omega_{v}\left(s\right)T_{ps}\left(s\right)-T_{\mathrm{pb}}\left(s\right)}{{1+G}_{a}\left(H_{d}\left(s\right)\omega_{d}\left(s\right)+H_{v}\left(s\right)\omega_{v}\left(s\right)s\right)T_{ps}\left(s\right)}.$$

Hence, the effects of *H_v_*(*s*) and *H_d_*(*s*) can be described by a sensitivity matrix *E*, which is given as follows

(8) $$Ε=\left(\begin{array}{cc} \frac{\partial T_{iso}}{\partial H_{v}} & \frac{\partial T_{iso}}{\partial H_{d}}\\ \frac{\partial T_{pos}}{\partial H_{v}} & \frac{\partial T_{pos}}{\partial H_{d}} \end{array}\right),$$

where

(9) $$\frac{\partial T_{iso}}{\partial H_{v}}=-\frac{G_{a}\left(T_{sb}\left(s\right)+G_{a}H_{d}\left(s\right)\omega_{d}\left(s\right)\right)\omega_{v}\left(s\right)T_{ps}{}^{2}\left(s\right)}{{\left(1+G_{a}\left(H_{d}\left(s\right)\omega_{d}\left(s\right)+H_{v}\left(s\right)\omega_{v}\left(s\right)s\right)T_{ps}\left(s\right)\right)}^{2}},$$

(10) $$\frac{\partial T_{iso}}{\partial H_{v}}=\frac{G_{a}\left(1+G_{a}H_{v}\left(s\right)\omega_{v}\left(s\right)T_{ps}\left(s\right)s-T_{pb}\left(s\right)\right)\omega_{d}\left(s\right)T_{ps}\left(s\right)}{{\left(1+G_{a}\left(H_{d}\left(s\right)\omega_{d}\left(s\right)+H_{v}\left(s\right)\omega_{v}\left(s\right)s\right)T_{ps}\left(s\right)\right)}^{2}},$$

(11) $$\frac{\partial T_{pos}}{\partial H_{v}}=\frac{G_{a}\left(T_{sb}\left(s\right)+G_{a}H_{d}\left(s\right)\omega_{d}\left(s\right)\right)\omega_{v}\left(s\right)T_{ps}{}^{2}\left(s\right)}{{\left(1+G_{a}\left(H_{d}\left(s\right)\omega_{d}\left(s\right)+H_{v}\left(s\right)\omega_{v}\left(s\right)s\right)T_{ps}\left(s\right)\right)}^{2}},$$

(12) $$\frac{\partial T_{pos}}{\partial H_{d}}=-\frac{G_{a}\left(1+G_{a}H_{v}\left(s\right)\omega_{v}\left(s\right)T_{ps}\left(s\right)s-T_{pb}\left(s\right)\right)\omega_{d}\left(s\right)T_{ps}\left(s\right)}{{\left(1+G_{a}\left(H_{d}\left(s\right)\omega_{d}\left(s\right)+H_{v}\left(s\right)\omega_{v}\left(s\right)s\right)T_{ps}\left(s\right)\right)}^{2}}.$$

From Equation (9) to Equation (12), one can obtain ∂*T_iso_*/∂*H_d_* = −∂*T_pos_*/∂*H_d_* and ∂*T_pos_*/∂*H_v_* = −∂*T_iso_*/∂*H_v_*. It means that the controllers have opposite effects on the AVI and positioning. The basic idea of the PFC is to shape the *H_d_*(*s*) and *H_v_*(*s*) with suitable *ω_d_*(*s*) and *ω_v_*(*s*), thus weakening the contradiction between the AVI and positioning in the frequency range.

## 3. Effects of AVI and Positioning Controls

Understanding the interaction law between the absolute velocity feedback (AVF) and the relative position feedback (RPF) is the basis for the design and optimization of PFC. To evaluate the effects of AVF and RPF, both the values of the *ω_d_*(*s*) and *ω_v_*(*s*) are firstly taken as 1, and the controllers are set as

(13) $$H_{d}\left(s\right)=kd_{p}+kd_{i}/s+kd_{d}s,\;H_{v}\left(s\right)=kv_{p}+kv_{d}s,$$

where *kd_p_*, *kd_i,_*, and *kd_d_* are the gains of *H_d_*(*s*), while *kv_p_* and *kv_d_* are the gains of *H_v_*(*s*). The parameters of the VIP system are listed in Table 1, which is consistent with the experimental setup described in Section 5.

### 3.1. Effect of AVF on AVI

The AVF is used to resist the external disturbance *x*_1_. When only the AVF is used, according to Equation (4) and Equation (5), the motion equation can be expressed as

(14) $$\left(m_{2}s^{2}+c_{2}s+k_{2}\right)x_{2}\left(s\right)-\left(c_{2}s+k_{2}\right)x_{1}\left(s\right)=-G_{a}H_{v}\left(s\right)sx_{2}\left(s\right).$$

Substituting Equation (13) into Equation (14), the vibration transmissibility can be written as

(15) $$T_{iso}^{v}\left(s\right)=\frac{x_{2}\left(s\right)}{x_{1}\left(s\right)}=\frac{x_{2}\left(s\right)s^{2}}{x_{1}\left(s\right)s^{2}}=\frac{c_{2}s+k_{2}}{\left(G_{a}kv_{d}+m_{2}\right)s^{2}+\left(G_{a}kv_{p}+c_{2}\right)s+k_{2}}.$$

**Figure 4:**
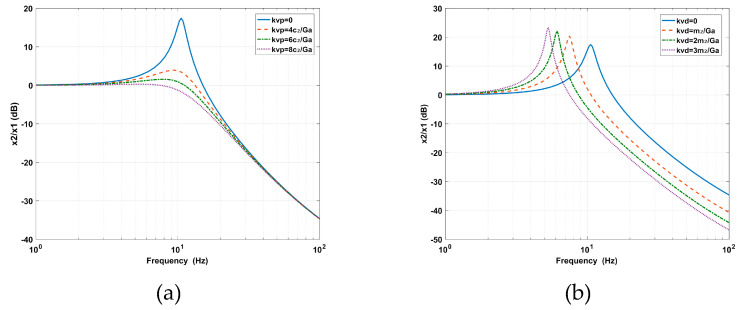
Effect of (**a**) the proportion *kvp* and (**b**) the derivative *kvd* of the absolute velocity feedback (AVF) on vibration transmissibility.

From the vibration transmissibility shown in Figure 4, it can be seen that the effect of *kv_p_* is equivalent to adding absolute damping to the supporting system, which attenuates the vibration amplitude around the inherent frequency. The effect of *kv_d_* is equivalent to the added virtual mass, which moves the inherent frequency forward and broadens the vibration isolation bandwidth, however, the vibration amplitude is amplified at the peak.

### 3.2. Effect of RPF on Positioning

When only the RPF is used, according to Equation (4) and Equation (5), the motion equation can be written as

(16) $$\left(m_{2}s^{2}+c_{2}s+k_{2}\right)x_{2}\left(s\right)-\left(c_{2}s+k_{2}\right)x_{1}\left(s\right)=G_{a}H_{d}\left(s\right)\delta D\left(s\right).$$

Because the base position cannot be obtained directly, the base acceleration is set as the input. Substituting Equation (13) into Equation (16), the deformation transmissibility is rewritten as

(17) $$T_{pos}^{d}\left(s\right)=\frac{\delta D\left(s\right)}{x_{1}\left(s\right)s^{2}}=\frac{x_{1}\left(s\right)-x_{2}\left(s\right)}{x_{1}\left(s\right)s^{2}}=\frac{m_{2}}{m_{2}s^{2}+\left(G_{a}kd_{d}+c_{2}\right)s+\left(k_{2}+G_{a}kd_{p}\right)+G_{a}kd_{i}∕s}.$$

**Figure 5:**
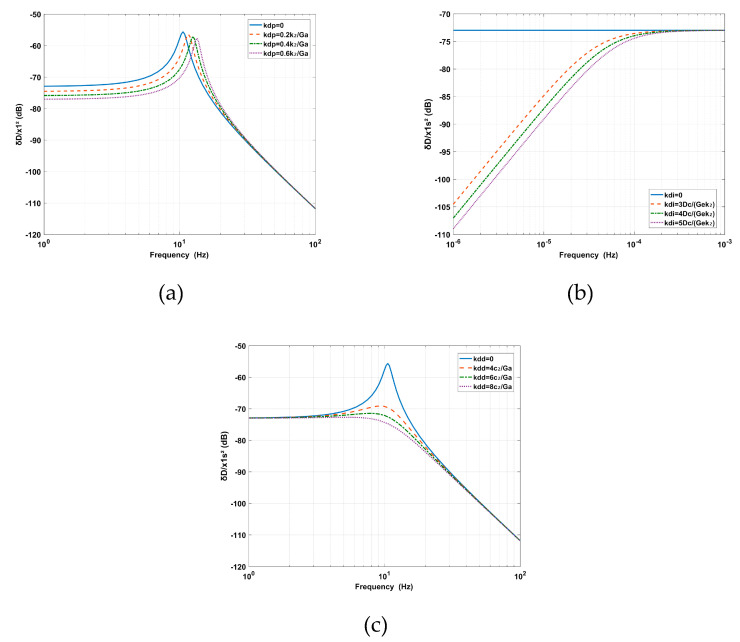
The effects of (**a**) the proportion *kdp*, (**b**) the integration *kdi* where *Ge* = 0.421 mm/V is the eddy-current sensor gain and *Dc* = 1 mm is the relative position between the payload and the base, (**c**) the effect of the derivative *kdd* of the relative position feedback (RPF) on the deformation transmissibility.

The influence of RPF on the deformation transmissibility is shown in Figure 5. The effect of *kd_p_* is equivalent to the mechanical stiffness parallel to *k*_2_, which can be used to increase the supporting stiffness. The addition of *kd_p_* increases the natural frequency and decreases the amplitude at low frequencies and the peak. The effect of *kd_i_* is equivalent to eliminating the steady-state error of the position and solving the leveling problem for the supporting system [26]. The amplitude is decreased at ultra-low frequencies with the addition of *kd_i_*. The effect of *kd_d_* is equivalent to adding relative damping between the base and the payload. With the addition of *kd_d_*, the amplitude around the inherent frequency is decreased gradually.

Additionally, it should be noted that the compliance is a way to evaluate the ability to resist the internal disturbance *F_d_*. As such, the compliance $C_{pos}^{d}\left(s\right)$ with the RPF is described as

(18) $$C_{pos}^{d}\left(s\right)=\frac{x_{2}\left(s\right)}{F_{d}}=\frac{1}{m_{2}s^{2}+\left(G_{a}kd_{d}+c_{2}\right)s+\left(k_{2}+G_{a}kd_{p}\right)+G_{a}kd_{i}∕s}.$$

The above equation has the same expression as $T_{pos}^{d}{(s)/m}_{2}$. This shows that the deformation transmissibility $T_{pos}^{d}\left(s\right)$ has a similar meaning to the compliance $C_{pos}^{d}\left(s\right)$.

### 3.3. Interaction of AVI and Positioning

When the AVF and the RPF are used simultaneously, the vibration transmissibility and the deformation transmissibility of the closed-loop system are

(19) $$T_{iso}^{vd}\left(s\right)=\frac{\left(c_{2}+G_{a}kd_{d}\right)s+\left(k_{2}+G_{a}kd_{p}\right)+G_{a}kd_{i}∕s}{\left(G_{a}kv_{d}+m_{2}\right)s^{2}+\left(G_{a}kv_{p}+G_{a}kd_{d}+c_{2}\right)s+\left(k_{2}+G_{a}kd_{p}\right)+G_{a}kd_{i}∕s},$$

(20) $$T_{pos}^{vd}\left(s\right)=\frac{\left(G_{a}kv_{d}+m_{2}\right)+G_{a}kv_{p}∕s}{\left(G_{a}kv_{d}+m_{2}\right)s^{2}+\left(G_{a}kv_{p}+G_{a}kd_{d}+c_{2}\right)s+\left(k_{2}+G_{a}kd_{p}\right)+G_{a}kd_{i}∕s}.$$

**Figure 6:**
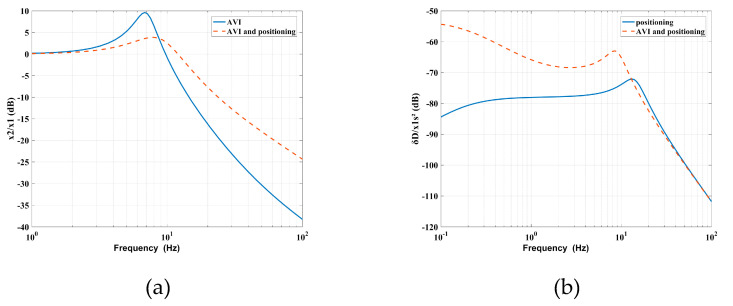
Contradictions between the AVF and RPF related to the (**a**) vibration transmissibility and (**b**) deformation transmissibility.

As shown in Figure 6a, the RPF adds the stiffness and relative damping between the base and the payload. The added stiffness increases the natural frequency, which narrows the vibration isolation bandwidth, while the relative damping decreases the maximum value of vibration transmissibility at the cost of amplifying the vibration amplitude at high frequencies. In Figure 6b, the AVF adds the mass and absolute damping to the payload, which weakens the connection between the base and the payload, so that the amplitude of the deformation transmissibility at low frequencies is amplified, which in turn deteriorates the positioning performance; that is, the interaction between the AVI and positioning leads to a reduction of the vibration isolation performance at high frequencies and a reduction of the positioning accuracy at low frequencies.

## 4. Analysis and Optimization of PFC

**Figure 7:**
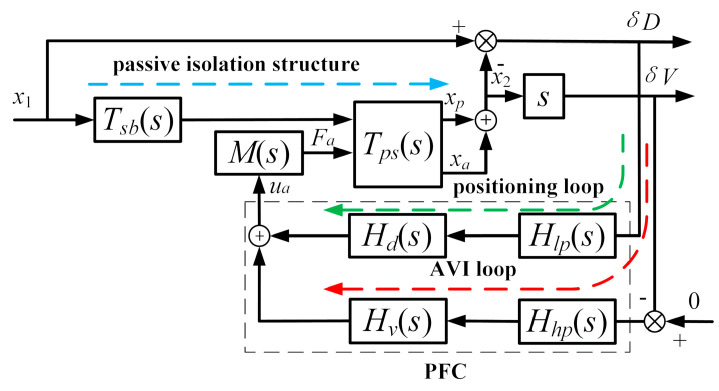
Block diagram of the PFC with the filter pair. The blue dotted line is the transfer path of the passive isolation structure, the green dotted line is the positioning loop, and the red dotted line is the AVI loop.

To suppress the effects of the interaction between the AVI and positioning on the frequency range, as shown in Figure 7, the *ω_v_*(*s*) and *ω_d_*(*s*) of the PFC adopt a low-pass filter *H_lp_*(*s*) and a high-pass filter *H_hp_*(*s*), respectively; that is, the PFC carries out the positioning at low frequencies and the AVI at high frequencies.

### 4.1. PFC Based on IOFs

**Figure 8:**
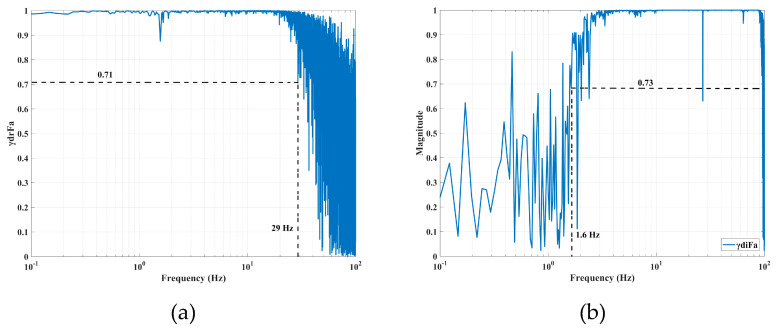
Coherence values between the signals of the actuator and the sensors: (**a**) XS4P18AB120 eddy-current sensor and (**b**) GS-11D-H velocity sensor.

To obtain a higher signal attenuation rate beyond the control band of the AVF or RPF, the conventional second-order IOFs are selected. The filters are as follows:

(21) $$H_{lp}\left(s\right)=\frac{1}{{\left(s/\left(2\pi f_{lp}\right)\right)}^{2}+2\zeta \left(s/\left(2\pi f_{lp}\right)\right)+1},$$

(22) $$H_{hp}\left(s\right)=\frac{{\left(s/\left(2\pi f_{hp}\right)\right)}^{2}}{{\left(s/\left(2\pi f_{hp}\right)\right)}^{2}+2\zeta \left(s/\left(2\pi f_{hp}\right)\right)+1},$$

where ζ is the damping ratio of the filters, which is selected as 0.7. Here, *f_lp_* and *f_hp_* are the cut-off frequency value for *H_lp_*(*s*) and *H_hp_*(*s*), respectively. As the control bandwidth of the AVF and RPF is limited by the sensor noise, the sensor bandwidth is firstly determined by the correlation function of the actuator and the sensor signals. In this paper, the relative position and the absolute velocity are measured by the XS4P18AB120 eddy-current position sensor and the GS-11D absolute velocity sensor, respectively. By exciting the VCM with white noise, the coherence values are obtained as shown in Figure 8. When the coherence values are greater than 0.7, the traditional control can be considered as a special PFC, with *f_lp_* = 29 Hz and *f_hp_* = 1.6 Hz.

To ensure the stability of the closed-loop system, the parameters of the controller are restricted by the gain margin and phase margin of the open-loop system. In practice, the phase margin should be greater than about 30° to prevent the system instability caused by the time delay, while the gain margin should be larger than 6 dB [27]. As shown in Figure 7, *x_p_* and *x_a_* are the payload movements caused by the passive isolation structure disturbance and the active control input, respectively. The relation between *x_p_* as the input and *x_a_* as the output is described as

(23) $$x_{a}\left(s\right)=G_{a}T_{ps}\left(s\right)H_{d}\left(s\right)H_{lp}\left(s\right)\left(x_{1}\left(s\right)-x_{p}\left(s\right)\right)-G_{a}T_{\mathrm{ps}}\left(s\right)H_{v}\left(s\right)H_{hp}\left(s\right)x_{p}\left(s\right)s.$$

Then, according to Equation (1) and Equation (23), the open-loop transfer function can be written as

(24) $$-\frac{x_{a}\left(s\right)}{x_{p}\left(s\right)}=G_{a}T_{ps}\left(s\right)\left(\left(-\frac{1}{T_{pb}\left(s\right)}+1\right)H_{d}\left(s\right)H_{lp}\left(s\right)+H_{v}\left(s\right)H_{hp}\left(s\right)s\right).$$

**Figure 9:**
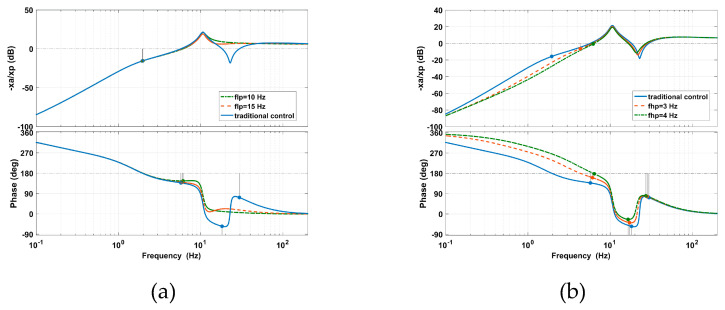
Open-loop system performance with the movement of (**a**) *flp* and (**b**) *fhp* caused by the PFC based on IOFs.

The gains of *H_v_*(*s*) and *H_d_*(*s*) are taken with moderate values as *kv_d_* = 2 *m*_2_*/G_a_*, *kv_p_ =* 8 *c*_2_*/G_a_*, *kd_p_ =* 0.5 *k*_2_*/G_a_*, *kd_i_ =* 3 *D_c_/*(*G_e_k*_2_), *kd_d_ =* 8 *c*_2_*/G_a_*. As shown in Figure 9, it can be seen that the adjustment of the cut-off frequencies will impact the gain margin and phase margin, meaning the gains of *H_v_*(*s*) and *H_d_*(*s*) should be reduced to ensure the system stability. The gain margins and phase margins according to different cut-off frequencies are listed in Table 2.

Furthermore, to evaluate the performance of the AVI and positioning, the vibration transmissibility and the deformation transmissibility of the closed-loop system are described as

(25) $$T_{iso}^{fdc}\left(s\right)=\frac{T_{pb}\left(s\right)+G_{a}T_{ps}\left(s\right)H_{d}\left(s\right)H_{lp}\left(s\right)}{1+G_{a}T_{ps}\left(s\right)H_{v}\left(s\right)H_{hp}\left(s\right)s+G_{a}T_{ps}\left(s\right)H_{d}\left(s\right)H_{lp}\left(s\right)},$$

(26) $$T_{pos}^{fdc}\left(s\right)=\frac{m_{2}T_{ps}\left(s\right)+G_{a}T_{ps}\left(s\right)H_{v}\left(s\right)H_{hp}\left(s\right)/s}{1+G_{a}T_{ps}\left(s\right)H_{v}\left(s\right)H_{hp}\left(s\right)s+G_{a}T_{ps}\left(s\right)H_{d}\left(s\right)H_{lp}\left(s\right)}.$$

**Figure 10:**
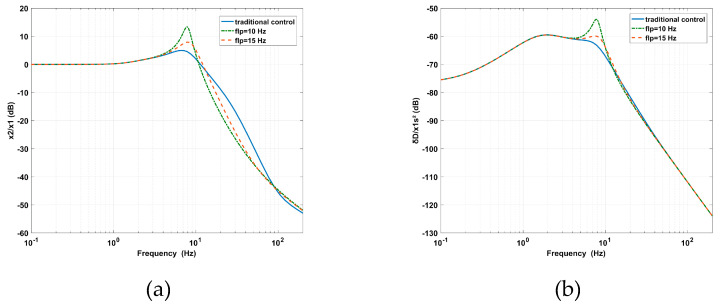
The effects of *flp* on the PFC based on IOFs for (**a**) vibration transmissibility and (**b**) deformation transmissibility.

As shown in Figure 10a, the decrease of *f_lp_* leads to the increase of the vibration transmissibility at the peak, the decease of the vibration transmissibility at high frequencies, and the enlargement of the AVI bandwidth. In Figure 10b, the amplitude of the deformation transmissibility at the peak is enlarged. This means that the virtual stiffness and relative damping are decreased.

**Figure 11:**
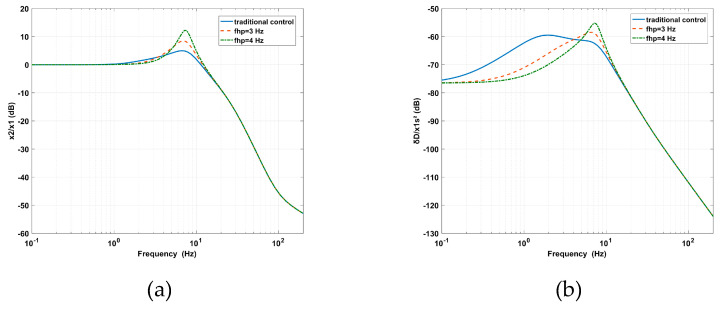
The effects of *fhp* on the PFC based on IOFs for (**a**) vibration transmissibility and (**b**) deformation transmissibility.

As shown in Figure 11a, with an increasing *f_hp_*, the amplitude of the vibration transmissibility at the peak is significantly amplified, and the AVI bandwidth is slightly narrowed. Due to the lower virtual mass and absolute damping, the effect of the AVF on the AVI is gradually reduced. In Figure 11b, by using a smaller virtual mass and less absolute damping, the position accuracy is strengthened at low frequencies. However, the amplitude of deformation transmissibility at the peak is enlarged due to the loss of absolute damping.

### 4.2. PFC Based on FOFs

The above analysis shows that the PFC based on IOFs can decrease the vibration transmissibility amplitude at high frequencies and improves the position accuracy at low frequencies; however, it amplifies the amplitude of the vibration and deformation transmissibility at the peak because of the loss of relative and absolute damping. Although the damping can be compensated by adding the gain from *kv_p_* and *kd_d_*, this results in system instability. Considering that FOFs can provide greater flexibility in optimizing both the magnitude and phase response than the IOF, a pair of FOFs is introduced into the PFC. The FOFs are considered as the generalized case of IOFs [28,29], for which are more flexible as they introduce more variables of fraction order, but also more complicated. To reduce the additional unknown parameters, the FOFs are formed by replacing the integral order of the IOFs with the fractional order, which can be described as

(27) $$H_{lp}^{fo}\left(s\right)=\frac{1}{{\left(s/\left(2\pi f_{lp}\right)\right)}^{\alpha_{1}}+2\zeta_{\alpha }{\left(s/\left(2\pi f_{lp}\right)\right)}^{\alpha_{2}}+1},$$

(28) $$H_{hp}^{fo}\left(s\right)=\frac{{\left(s/\left(2\pi f_{hp}\right)\right)}^{2}}{{\left(s/\left(2\pi f_{hp}\right)\right)}^{\beta_{1}}+2\zeta_{\beta }{\left(s/\left(2\pi f_{hp}\right)\right)}^{\beta_{2}}+1},$$

where *α*_1_, *α*_2_, *β*_1_, and *β*_2_∈R represent the fractional order.

The design specifications required for the system stability and performance are as follows:

(1) Phase margin = *PM* ≥ 40°;
(2) Gain margin = *GM* ≥ 15 dB;
(3) Vibration transmissibility: max(${\left|T_{iso}^{fdc}\left(s\right)\right|}_{dB}$) ≤ 5 dB and the amplitude crossover frequency is close to 10 Hz;
(4) Positioning transmissibility: max(${\left|T_{pos}^{fdc}\left(s\right)\right|}_{dB}$) ≤ −75 dB when the frequency is smaller than 2 Hz, otherwise max (${\left|T_{pos}^{fdc}\left(s\right)\right|}_{dB}$) ≤ −60 dB.

To simplify the parameter searching process for the optimization of controllers and filters, the damping ratios of filters *ζ_α_* and *ζ_β_* are set as 0.7; both the variation range of *α*_2_ and *β*_2_ is from 0 to 2 and the relations between *α*_1_ and *α*_2_, and between *β*_1_ and *β*_2_ are set as *α*_1_ = 2*α*_2_ and *β*_1_ = 2*β*_2_, respectively. Because *kd_i_* affects the deformation transmissibility at ultralow frequencies, it is not considered in the optimization process. The Matlab optimization toolbox is applied to find the minimum of the constrained nonlinear multivariable function. The optimized controller gains and the Fractional-Order Filters are

(29) $$H_{d}^{fo}\left(s\right)=8k_{2}/G_{a}+3D/\left(G_{e}k_{2}s\right)+10.23c_{2}s/G_{a},$$

(30) $$H_{v}^{fo}\left(s\right)=10c_{2}/G_{a}+2.5m_{2}s/G_{a},$$

(31) $$H_{lp}^{fo}\left(s\right)=\frac{1}{{\left(s/\left(20\pi \right)\right)}^{3}+1.4{\left(s/\left(20\pi \right)\right)}^{1.5}+1},$$

(32) $$H_{hp}^{fo}\left(s\right)=\frac{{\left(s/\left(10\pi \right)\right)}^{3.6}}{{\left(s/\left(10\pi \right)\right)}^{3.6}+1.4{\left(s/\left(10\pi \right)\right)}^{1.8}+1}.$$

The FOF provides more flexibility and insight in the filter design, but the ideal FOF can only be realized by proper approximation with the IOF [30]. The Oustaloup filter approximation is widely used in fractional calculus [31], and it is adopted in the implementation of FOFs in Equations (30) and (31). An Oustaloup filter can be designed as

(33) $$s^{\gamma }=k\overset{N}{\underset{n=1}{\prod }}\frac{s+\omega_{k}^{\prime }}{s+\omega_{k}},$$

where *γ* is the order of the derivative and *N* is the order of the filter. The poles, zeros, and gains are calculated from

(34) $$\begin{array}{ll} \omega_{k}^{\prime }= & \omega_{b}\omega_{u}^{\left(2n-1-\gamma \right)/N},\\ \omega_{k}= & \omega_{b}\omega_{u}^{\left(2n-1+\gamma \right)/N},\\ k= & \omega_{h}^{\gamma }, \end{array}$$

with $\omega_{u}=\sqrt{\omega_{h}/\omega_{b}}$, where *ω_b_* and *ω_h_* are the lower and upper limits of the frequency range for the Oustaloup filter.

The designed FOFs can be approximated more accurately with a higher-order filter. However, this costs more computing time and may distort the input signal. Therefore, the fractional-order derivatives *s*^0.5^ and *s*^0.8^ are approximated by the 4-order and 2-order Oustaloup filters, respectively, as *ω_b_* and *ω_h_* are determined using the related AVI and positioning bandwidth range, i.e., 1 Hz to 50 Hz. From *ω_b_* = 0.314 rad/sec and *ω_a_* = 2.51 × 10^3^ rad/sec, $H_{lp}^{fo}\left(s\right)$ and $H_{hp}^{fo}\left(s\right)$ are obtained as follows:

(35) $$Ho_{lp}^{fo}\left(s\right)=\frac{2.4805\times 10^{5}\left(s+1433\right)\left(s+151.5\right)\left(s+16.02\right)\left(s+1.694\right)}{\left(s+1415\right)\left(s+18.92\right)\left(s+1.701\right)\left(s^{2}+169s+8150\right)\left(s^{2}-2.332s+3939\right)},$$

(36) $$Ho_{hp}^{fo}\left(s\right)=\frac{s^{2}{\left(s+44.04\right)}^{2}{\left(s+0.4924\right)}^{2}}{\left(s^{2}+42.06s+493.5\right)\left(s^{2}+61.15s+1477\right)\left(s^{2}-12.83s+1009\right)}.$$

**Figure 12:**
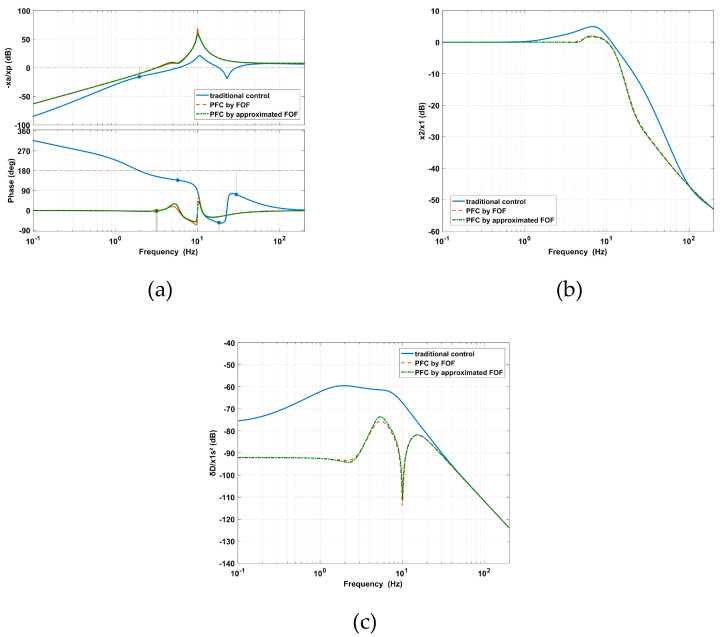
Comparisons between the traditional control and PFC based on FOFs regarding the (**a**) open-loop system, (**b**) vibration transmissibility, and (**c**) deformation transmissibility.

In Figure 12, it can be seen that the approximations of the fractional-order derivatives *s*^0.5^ and *s*^0.8^ are suitable through comparing the theoretical and approximated bode diagrams. Moreover, as shown in Figure 12a, the PFC based on FOFs results in a phase margin of 179° at 3.21 Hz and provides more stability than the traditional control. As shown in Figure 12b,c, the PFC based on FOFs can simultaneously decrease the amplitude of the vibration transmissibility at high frequencies and the deformation transmissibility at low frequencies. This shows that the amplitudes of the vibration and deformation at the peak are restrained because the FOFs provide a larger gain range for *H_v_*(*s*) and *H_d_*(*s*) to coordinate the AVI and positioning.

## 5. Experimental Verification

### 5.1. Experimental Setup

**Figure 13:**
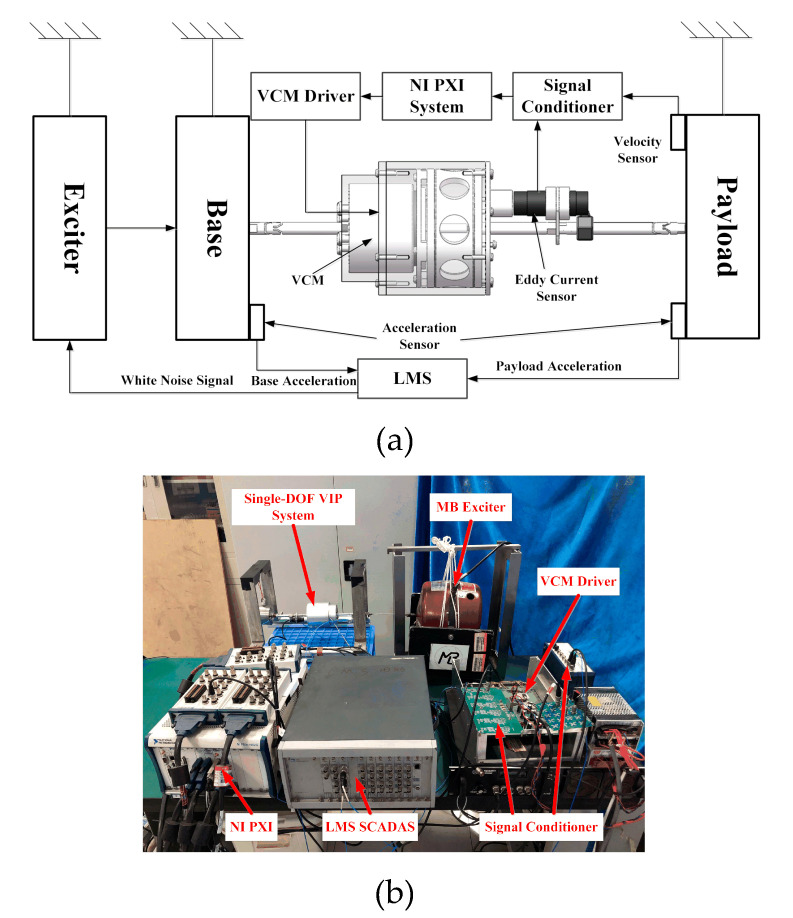
The experimental setup: (**a**) schematic diagram and (**b**) photograph.

As shown in Figure 13, the experimental system was established to verify the PFC. It can be seen that the single-DOF VIP system and the exciter are hung on the frame to avoid additional stiffness and damping along the motion direction. The two mass blocks corresponding to the payload and the base are connected by the supporting unit through two flexure hinges. This ensures that the experimental system is only affected by the axial force. The NI (National Instruments) PXI (Peripheral Component Interconnection extensions for Instrumentation) real-time system collects the feedback signals from the velocity sensor and eddy-current sensor through the PXI-6123 acquisition card and controls the VCM to actuate the VIP system. The LMS (Leuven Measurement & System) SCADAS (Supervisory Control And Data Acquisition) system controls the MB Dynamic MODAL110 instrument to simulate the base excitation and analyzes the control performance according to the vibration signals of the base and the payload, which are collected by the PCB356A17 acceleration sensor.

### 5.2. Experimental Results

**Figure 14:**
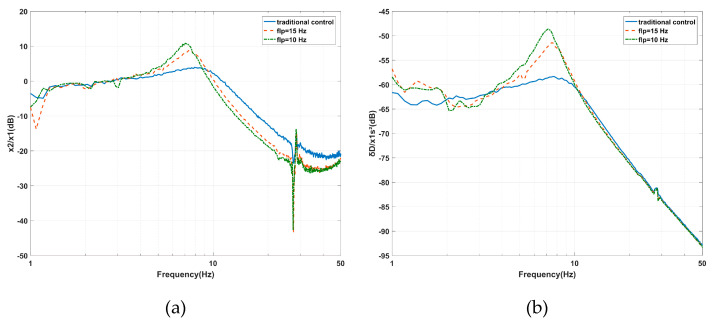
Experimental results of the effects of the *flp* effects on the PFC based on IOFs regarding the (**a**) vibration transmissibility and (**b**) deformation transmissibility.

The experimental analyses of *f_hp_* and *f_lp_* were carried out. The decrease of *f_lp_* from 29 to 10 Hz due to the effect of *f_lp_* on the AVI is shown in Figure 14a. The maximum value of the vibration transmissibility increased from 3.93 dB at 7.91 Hz to 10.84 dB at 7.03 Hz. The cross-frequency (where the amplitude of vibration transmissibility is 0 dB) decreased from 11.23 to 9.57 Hz. The performance was improved at high frequencies but deteriorated from 3.32 to 8.7 Hz as *f_lp_* is equal to 10 Hz and from 3.5 to 9.3 Hz as *f_lp_* is equal to 15 Hz. In Figure 14b, as the same tendency for *f_lp_* changes, the maximum value of the deformation transmissibility is enlarged from −58.37 dB at 7.72 Hz to −48.7 dB at 7.23 Hz. The amplitude amplified in the frequency range of 3.6 to 10.45 Hz as *f_lp_* is equal to 10 Hz, while the amplitude amplified in the frequency range of 3.9 to 10.84 Hz as *f_lp_* is equal to 15 Hz. The experimental results are consistent with Figure 10.

**Figure 15:**
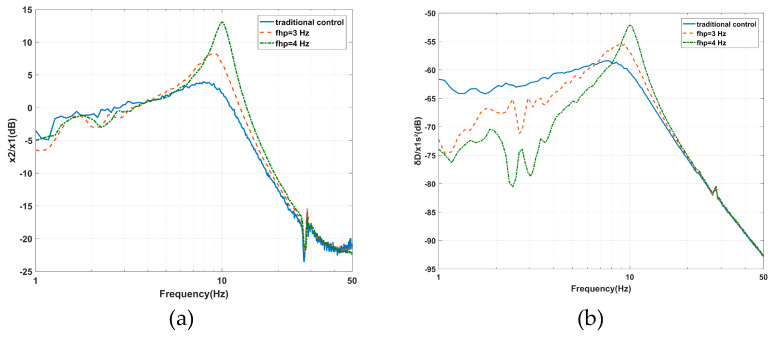
Experimental results of the effects of the *fhp* on the PFC based on IOFs regarding the (**a**) vibration transmissibility and (**b**) deformation transmissibility.

The increase of *f_hp_* from 1.6 to 4 Hz due to the effect of *f_hp_* on the AVI is shown in Figure 15a. The maximum value of the vibration transmissibility is amplified from 3.93 dB at 7.91 Hz to 10.06 dB at 13.07 Hz, while the cross-frequency moves from 11.23 to 13.57 Hz. The amplitudes are amplified in the frequency range of 5.47 to 27.05 Hz because *f_hp_* is equal to 3 Hz and from 4.2 to 27.05 Hz because *f_hp_* is equal to 4 Hz. The variation trend is consistent with Figure 11a. The influence of *f_hp_* on positioning is shown in Figure 15b. The maximum value of the deformation transmissibility is amplified from –58.37 dB at 7.72 Hz to –52.15 dB at 10.06 Hz. The amplitude decreases before 6.64 Hz as *f_hp_* is equal to 3 Hz and before 8.11 Hz as *f_hp_* is equal to 4 Hz. The amplitude is amplified around the inherent frequency and diminished at low frequencies, similar to that shown in Figure 11b. The results of *f_lp_* and *f_hp_* in terms of the effects on the performance of the AVI and positioning are listed in Table 3.

The performance of the AVI at high-frequencies and the positioning at low frequencies can be improved by the PFC based on IOFs, however, both the amplitudes of the vibration transmissibility and deformation transmissibility are undesirably amplified around the inherent frequency. To solve this problem, the $H_{lp}^{fo}\left(s\right)$ and $H_{hp}^{fo}\left(s\right)$ filters of the PFC based on FOFs are introduced and the corresponding experiments are carried out. By setting *f_lp_* as 10 Hz and *f_hp_* as 5 Hz, as calculated in Section 4.2, the PFC based on FOFs is compared with the traditional control and the PFC based on IOFs.

**Figure 16:**
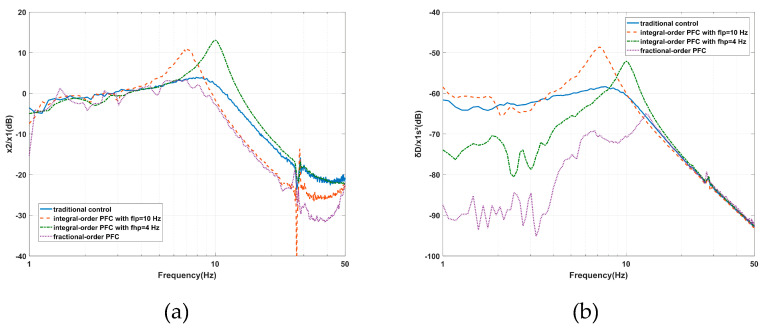
Experimental results of the effects of the PFC based on FOFs on the: (**a**) vibration transmissibility and (**b**) deformation transmissibility.

As shown in Figure 16a, the PFC based on FOFs moves the cross-frequency to 8.6 Hz and diminishes the maximum value to 3.3 dB at 6.93 Hz for the vibration transmissibility. Compared with the traditional control and the PFC based on IOFs with a *fhp* value equal to 4 Hz, the PFC based on FOFs improves the AVI performance from 6.54 Hz to the end of the frequency range. It seems that the vibration transmissibility at high frequencies is moved forward. The PFC based on IOFs with an *flp* value equal to 10 Hz can also achieve this effect at the cost of amplifying the amplitude around the inherent frequency. The AVI performance is improved by the limited control bandwidth of the positioning and the increased gains of *kv_p_* and *kv_d_*. These targets can be realized by the PFC based on FOFs simultaneously, which is not possible with the PFC based on IOFs.

As shown in Figure 16b, the PFC based on FOFs restrains the maximum value to –64.96 dB at 12.79 Hz for the deformation transmissibility. Compared with the traditional control and the PFC based on IOFs with an *flp* value equal to 10 Hz, the deformation performance is significantly improved when the frequency is less than 12.7 Hz. The PFC based on IOFs with an *fhp* value equal to 4 Hz can also enhance the positional stability at low frequencies; however, not to the same degree as with the PFC based on FOFs. The amplitude around the inherent frequency is also amplified. The PFC based on FOFs improves the positioning performance by limiting the control bandwidth of the AVI and increasing the gains of *kd_p_* and *kd_d_*. The deformation transmissibility starts to increase from about 10 Hz due to the control bandwidth of positioning being limited by the $H_{lp}^{fo}\left(s\right)$ filter.

Significantly, the PFC based on FOFs suppresses the peak amplitude obviously in the natural frequency area, which cannot be avoided when using the IOFs, while the performance of AVI at high frequencies and positioning at low frequencies is also guaranteed.

### 5.3. Discussion

Three different control strategies are presented in this section, and the control effects are evaluated based on the vibration transmissibility and deformation transmissibility. Compared with the traditional control, the low-frequency positioning using the PFC based on IOFs improves the AVI performance obviously at high frequencies. Both the amplitudes of the vibration transmissibility and deformation transmissibility around the inherent frequency are increased. The attenuation of the RPF via low-frequency positioning is the primary cause of this change. The high-frequency AVI by the PFC based on IOFs improves the positioning performance notably at low frequencies. This introduces the same adverse effects for the peak amplitudes of the vibration transmissibility and deformation transmissibility as the low-frequency positioning does. The requirements for the AVI and positioning are not satisfied by the PFC based on IOFs.

The PFC based on FOFs can overcome the above limitation. The performance of the AVI at low frequencies and the positioning at high frequencies is improved, and both of the peak amplitudes are decreased. The FOFs provide more parameters that can be adjusted, which increases the flexibility of the controller. This gives more choices for the cut-off of the low-frequency positioning and high-frequency AVI, and higher feedback gains for coordination of the AVI and positioning performance.

## 6. Conclusions

This paper proposes a novel PFC strategy based on FOFs. The PFC can weaken the interaction between the AVI and positioning by using low-frequency positioning and high-frequency AVI. The PFC is verified using a single-DOF supporting system. Compared with the traditional control, the PFC based on IOFs can decrease the amplitude of the vibration transmissibility at high frequencies and the deformation transmissibility at low frequencies. However, both of the amplitudes around the inherent frequency are amplified and the adjustment of the cut-off frequency decreases the stability margin. Thus, the performance of the AVI and positioning cannot be further improved by the limited control gains. The PFC based on FOFs is optimized to overcome this contradiction between the system stability and the VIP performance. Compared with the traditional control, both the maximum value of the vibration and deformation transmissibility are restrained, while the performance of the AVI and positioning is improved in the frequency range from 6.54 Hz to the end and at less than 12.7 Hz, respectively.

## Figures and Tables

**Table 1:** Parameters of single-DOF supporting system.

Symbol	Property	Value
*m*_1_	Mass of payload	2 kg
*m*_2_	Mass of base	2.2 kg
*k*_2_	Stiffness constant	9.59 KN/m
*c*_2_	Damping coefficient	20 Ns/m
*f*	Inherent frequency	10.5 Hz
*Ga*	Voice coil motor (VCM) gain	3.17 N/V
*Ge*	Eddy-current sensor gain	0.421 mm/V

**Table 2:** Gain margins and phase margins by different cut-off frequencies.

Cut-Off Frequency	Gain Margin and Phase Crossover Frequency	Phase Margin and Gain Crossover Frequency
*flp* = 10 Hz & *fhp* = 1.6 Hz	15.5 dB @ 1.98 Hz	34.2° @ 6.11 Hz
*flp* = 15 Hz & *fhp* = 1.6 Hz	15.6 dB @ 1.96 Hz	40.3° @ 5.96 Hz
*flp* = 29 Hz & *fhp* = 1.6 Hz (Traditional control)	15.7 dB @ 1.95 Hz	42.6° @ 5.74 Hz 125° @ 18.2 Hz 108° @ 29.5 Hz
*flp* = 29 Hz & *fhp* = 3 Hz	6.42 dB @ 4.33 Hz	19.5° @ 6.1 Hz 141° @ 17.2 Hz 103° @ 28.2 Hz
*flp* = 29 Hz & *fhp* = 4 Hz	0.734 dB @ 6.21 Hz	2.33° @ 6.41 Hz 156° @ 16.5 Hz 99.6° @ 27.2 Hz

**Table 3:** The performance of AVI and positioning with different *f_lp_* and *f_hp_* values.

Cut-Off Frequency	AVI Performance	Positioning Performance
*flp* = 10 Hz & *fhp* = 1.6 Hz	Maximum value: 10.84 dB @ 7.03 Hz Cross-frequency: 9.57 Hz	Maximum value: −48.7 dB @ 7.22 Hz
*flp* = 15 Hz & *fhp* = 1.6 Hz	Maximum value: 8.89 dB @ 7.52 Hz Cross-frequency: 10.16 Hz	Maximum value: −51.41 dB @ 7.52 Hz
*flp* = 29 Hz & *fhp* = 1.6 Hz (Traditional control)	Maximum value: 3.93 dB @ 7.91 Hz Cross-frequency: 11.23 Hz	Maximum value: −58.37 dB @ 7.72 Hz
*flp* = 29 Hz & *fhp* = 3 Hz	Maximum value: 8.98 dB @ 8.24 Hz Cross-frequency: 12.5 Hz	Maximum value: −55.36 dB @ 8.89 Hz
*flp* = 29 Hz & *fhp* = 4 Hz	Maximum value: 10.06 dB @ 13.07 Hz Cross-frequency: 13.57 Hz	Maximum value: −52.15 dB @ 10.06 Hz

## References

[1] Ito S., Schitter G. (2015). Comparison and classification of high-precision actuators based on stiffness influencing vibration isolation. IEEE-Asme Trans. Mech..

[2] Butler H. (2011). Position control in lithographic equipment. IEEE Contr. Syst. Mag..

[3] Kienholz D.A. Active alignment and vibration control system for a large airborne optical system. Proceedings of the SPIE-The International Society for Optical Engineering.

[4] Heertjes M., de Graaff K., van der Toorn J.G. (2005). Active vibration isolation of metrology frames; a modal decoupled control design. J. Vib. Acoust..

[5] Tjepkema D., Dijk J.V., Soemers H.M.J.R. (2012). Sensor fusion for active vibration isolation in precision equipment. J. Sound. Vib..

[6] Gong Z.P., Ding L., Yue H.H., Gao H.B., Liu R.Q., Deng Z.Q., Lu Y.F. (2019). System integration and control design of a maglev platform for space vibration isolation. J. Vib. Control.

[7] Ma K., Ghasemi-Nejhad M.N. (2004). Frequency-weighted adaptive control for simultaneous precision positioning and vibration suppression of smart structures. Smart Mater Struct..

[8] van Dijk J. (2009). Mechatronic design of hard-mount concepts for precision equipment. Motion and Vibration Control.

[9] Pu H.Y., Li J.M., Wang M., Huang Y.N., Sun Y. (2019). Optimum design of an eddy current damper considering the magnetic congregate effect. J. Phys. D Appl. Phys..

[10] Pu H.Y., Yuan S.J., Peng Y., Meng K., Zhao J.L., Xie R.Q., Huang Y.N., Sun Y., Yang Y., Xie S.R. (2019). Multi-layer electromagnetic spring with tunable negative stiffness for semi-active vibration isolation. Mech. Syst. Signal Pr..

[11] Hanieh A.A. (2003). Active isolation and damping of vibrations via Stewart platform. Ph.D. Thesis.

[12] van der Poel G.W. (2010). An exploration of active hard mount vibration isolation for precision equipment. Ph.D. Thesis.

[13] Liao F.H., Li X.P., Yuan Z.Y. (2012). Self-tuning of position feedback and velocity feedback of active vibration isolation system with 6 dofs. Mechanika.

[14] Beijen M.A., Heertjes M.F., Butler H., Steinbuch M. (2016). Practical tuning guide to mixed feedback and feedforward control of soft-mounted vibration isolators. IFAC-PapersOnLine.

[15] Zhang Y.K., Dong W.B., Liu W., Li Z.F., Lv S.M., Sang X.R., Yang Y. (2017). Verification of the microgravity active vibration isolation system based on parabolic flight. Microgravity Sci. Technol..

[16] Jackson M., Kim Y., Whorton M. Design and Analysis of the g-LIMIT Baseline Vibration Isolation Control System. Proceedings of the AiAA Guidance, Navigation, & Control Conference & Exhibit.

[17] Yang X.L., Wu H.T., Chen B., Kang S.Z., Cheng S.L. (2019). Dynamic modeling and decoupled control of a flexible stewart platform for vibration isolation. J. Sound Vib..

[18] Yun Y., Li Y. (2012). Modeling and control analysis of a 3-pupu dual compliant parallel manipulator for micro positioning and active vibration isolation. J. Dyn. Syst-T. Asme..

[19] Zhu W.H., Tryggvason B., Piedboeuf J.C. (2006). On active acceleration control of vibration isolation systems. Control Eng. Pract..

[20] Matichard F., Lantz B., Mason K., Mittleman R., Abbott B., Abbott S., Allwine E., Barnum S., Birch J., Biscans S. (2015). Advanced LIGO two-stage twelve-axis vibration isolation and positioning platform. Part 1: Design and production overview. Precis. Eng..

[21] Thayer D., Campbell M., Flotow A.V., Vagners J. (2012). Six-axis vibration isolation system using soft actuators and multiple sensors. J. Spacecr. Rocket..

[22] Coopmans C., Jensen A.M., Chen Y.Q. (2014). Fractional-order complementary filters for small unmanned aerial system navigation. J. Intell. Robot Syst..

[23] Luo Y., Zhang T., Zhou L., Chen Y.Q. (2015). Pre-filtering and head-dependent adaptive feed-forward compensation for translation vibration in hard-disc-drive. Mechatronics.

[24] Charef M., Charef A. Fractional order controller based on the fractionalization of PID controller. Proceedings of the 2017 5th International Conference on Electrical Engineering-Boumerdes (ICEE-B).

[25] Zheng W.J., Luo Y., Wang X.H., Pi Y.G., Chen Y.Q. (2017). Fractional order pi^λ^d^μ^ controller design for satisfying time and frequency domain specifications simultaneously. ISA Trans..

[26] Fleming A.J. (2010). Nanopositioning system with force feedback for high-performance tracking and vibration control. IEEE-Asme. T. Mech..

[27] Skogestad S., Postlethwaite I. (2001). Multivariable Feedback Control: Analysis and Design.

[28] Soltan A., Radwan A.G., Soliman A.M. (2012). Fractional order filter with two fractional elements of dependent orders. Microelectron. J..

[29] Radwan A.G., Elwakil A.S., Soliman A.M. (2009). On the generalization of second-order filters to the fractional-order domain. J. Circuit Syst. Comp..

[30] Ma C., Hori Y. (2007). Fractional-order control: Theory and applications in motion control [past and present]. IEEE Ind. Electron. Mag..

[31] Oustaloup A., Levron F., Mathieu B., Nanot F.M. (2002). Frequency-band complex noninteger differentiator: Characterization and synthesis. IEEE Trans. Circuits Syst. I.

